# Pillar[5]arene Based [1]rotaxane Systems With Redox-Responsive Host-Guest Property: Design, Synthesis and the Key Role of Chain Length

**DOI:** 10.3389/fchem.2019.00508

**Published:** 2019-07-23

**Authors:** Runmiao Zhang, Chenwei Wang, Renhua Long, Tingting Chen, Chaoguo Yan, Yong Yao

**Affiliations:** ^1^College of Chemistry, Nantong University, Nantong, China; ^2^College of Chemistry, Yangzhou University, Yangzhou, China

**Keywords:** pillar[n]arenes, rotaxanes, electrochemically reversible, single-crystal X-ray, ferrocene

## Abstract

Pillar[*n*]arenes are a new type of macrocyclic compounds, which were first reported in 2008 by Ogoshi. They not only have cylindrical, symmetrical, and rigid structures, but also have many advantages, including easy functionalization and rich host-guest properties. On the other hand, mechanically interlocked molecules (MIMs) exist extensively in nature which have been artificially synthesized and widely applied in the fields of nanotechnology and biology. Although pillar[5]arene-based MIMs have been investigated much over recent years, pillar[5]arene-based [1]rotaxanes are very limited. In this report, we synthesized a series of amide-linked pillar[5]arene-based [1]rotaxanes with ferrocene unit as the stopper. Under the catalysis of HOBT/EDCL, the mono-amido-functionalized pillar[5]arenes were amidated with ferrocene carboxylic acid to constructed ferrocene-based [1]rotaxanes, respectively. The structure of the [1]rotaxanes were characterized by ^1^H NMR, ^13^C NMR, 2D NMR, mass spectroscopy, and single-crystal X-ray structural determination. In the experiment, the monofunctionalized pillar[5]arene was synthesized with a self-inclusion property, which allows for forming a pseudo-rotaxane. The key role is the length of the imine chain in this process. The formation of a rotaxane was realized through amidation of ferrocene dicarboxylic acid, which acted as a plug. In addition, due to the ferrocene units, the pillar[5]arene-based [1]rotaxanes perform electrochemically reversible property. Based on this nature, we hope these pillar[5]arene-based [1]rotaxanes can be applied in battery devices in the future.

## Introduction

Mechanically interlocked molecules (MIMs) are a type of “star” molecule due to their beautiful and interesting architectures and wide applications in the area of biology and nanoscience (Bissell et al., 1994; Brouwer et al., 2001; Zhu and Chen, 2005; Crowley et al., 2009; Yonath, 2010; Zhang et al., 2011; Li et al., 2014; Wang et al., 2015, 2018). Among various MIMs, rotaxanes, which have dumbbell-like structures with a wheel sliding along an axle, have attracted great interest due to their wide application in preparation of artificial molecular machines (Green et al., 2007; Lewandowski et al., 2013; Zhang et al., 2013). [1]rotaxanes, whose wheels and axles are connected in one molecule by covalent bonds, have a stable threaded form in both solution and solid state (Hiratani et al., 2004; Franchi et al., 2008; Li et al., 2012). However, the efficient synthesis of [1]rotaxanes is very difficult due to their subtle structure. To the best of our knowledge, there are very limited studies about the synthesis and properties of macrocycle based [1]rotaxanes. For example, Prof. Yang et al. prepared a functionalized [1]rotaxane and applied it to catalysis Knoevenagel reaction in CHCl_3_ (Du et al., 2017). Prof. Qu et al. fabricated a novel [1]rotaxane-based molecular motion modified with ferrocene groups (Li et al., 2012).

Pillar[*n*]arenes (Ogoshi et al., 2008; Cragg and Sharma, 2012; Xue et al., 2012; Si et al., 2015; Wang et al., 2015, 2016; Sun et al., 2018; Xiao and Wang, 2018; Xiao et al., 2019a,b), which are the newest host compounds in supramolecular chemistry after crown ethers (Liu et al., 2017; Yoo et al., 2019), cyclodextrins (Fu et al., 2019), calix[*n*]arenes (Dalgarno et al., 2007), and cucurbiturils (Murray et al., 2017), have attracted extensive investigations due to their pillar-like topology, rigid structures, electron-rich cavities, and rich host-guest properties (Song and Yang, 2015; Li et al., 2019; Wang et al., 2019). Up to now, pillar[*n*]arene-based pseudo[1]rotaxanes with ammonium units, urea groups, pyridinium salt or biotin units as the axles have been investigated a lot (Strutt et al., 2012; Ni et al., 2014; Wu et al., 2015), but the further formation of [1]rotaxanes is difficult due to the lack of reactivity with stoppers. With the constant efforts by scientists, several examples of pillar[*n*]arene-based [1]rotaxanes have been fabricated successfully. For example, Prof. Xue et al. combined C-H·π and ion-pair interactions to construct a pillar[5]arene-based [1]rotaxane in a yield of 73% (Xia and Xue, 2014). Prof. Yan et al. prepared a series of pillar[5]arene-based [1]rotaxanes from mono-amide-modified pillar[5]arenes with different lengths of the axles (Han et al., 2016).

Herein, we designed and synthesized a series of pillar[5]arene-based [1]rotaxanes with *N*-aminoalkyl amides as the axles and ferrocenecarboxylic acid as the stoppers through a method called “threading-followed-by-stoppering” (Cao et al., 2000). Self-included pillar[5]arene-based pseudo[1]-rotaxanes **P[5]^n^PRs** were prepared from monoester modified copillar[5]arene according previous research. Then pillar[5]arene based [1]rotaxanes **P[5]^n^Rs** were directly obtained by **P[5]^n^PRs** reacted with ferrocenecarboxylic acid as the stopper under the catalysis of HOBT/EDCL. Importantly, we found that the length of *N*-aminoalkyl chains play a key role in the formation of [1]rotaxanes—only when the number of carbon on the *N*-aminoalkyl chains larger than three can it form [1]rotaxanes. Moreover, these [1]rotaxanes showed electrochemically reversible properties due to the ferrocene unit on them.

## Materials and Methods

### Synthesis of Pillar[5]arenes-Based [1]rotaxanes and Mono-ferrocene Modified Pillar[5]arene

**Scheme 1:**
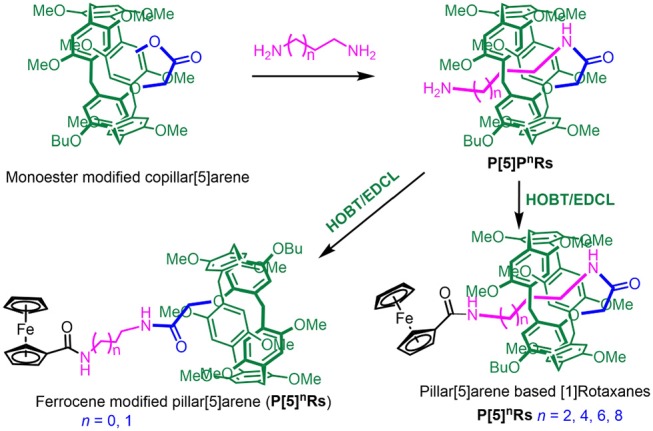
Synthetic route to a series of pillar[5]arene based [1]rotaxanes.

Based on previous work (Han et al., 2016), **P[5]^n^PRs** were obtained directly from mono-ester modified pillar[5]arene (Scheme 1). Then, **P[5]^n^Rs** and mono-ferrocene modified pillar[5]arene were successfully synthesized by **P[5]^n^PRs** reacted with ferrocene-carboxylic acid as the stopper under the catalysis of HOBT/EDCL. We take when *n* = 4 as a model reaction, **P[5]**^**4**^**PR** (0.203 g, 0.2 mmol), ferrocenecarboxylic acid (0.052 g, 0.2 mmol), HOBT(0.038 g, 0.25 mmol), and EDCL (0.055 g,0.25 mmol) were stirred in 10 mL dry CHCl_3_ over night at room temperature. The reaction solvent was evaporated and the residue was purified by flash column chromatography on silica gel (CH_2_Cl_2_/CH_3_OH, *v*/*v* 15:1) to give **P[5]**^**4**^**R** as a yellow solid (0.195 g). Other **P[5]^n^PRs** and mono-ferrocene modified pillar[5]arene were prepared with the similar method (Scheme 1).

#### P[5]^2^R

Yellow solid, 78.6%, m.p. 106.9-108.5°C; ^1^H NMR (400 MHz, CDCl_3_) δ: 7.05–6.89 (m, 7 H, ArH), 6.84 (d, *J* = 2.5 Hz, 1 H, ArH), 6.80 (s, 1 H, ArH), 6.60 (s, 1 H, ArH), 5.04–4.81 (m, 4 H, CH_2_), 4.50 (s, 2 H, ArH), 4.39 (s, 2 H, ArH), 4.24 (d, *J* = 2.5 Hz, 5 H, ArH), 4.05–3.95 (m, 2 H, CH_2_), 3.95–3.60 (m, 32 H, 24 CH_3_, 8 CH_2_), 3.54 (s, 4 H, CH_2_), 1.80 (d, *J* = 8.1 Hz, 2 H, CH_2_), 1.55 (d, *J* = 7.6 Hz, 2 H, CH_2_), 1.02 (d, *J* = 7.5 Hz, 3 H, CH_2_), −1.90 (d, *J* = 50.7 Hz, 2 H, CH_2_), −2.19 (d, *J* = 42.0 Hz, 2 H, CH_2_); ^13^C NMR (101 MHz, CDCl_3_) (Figure S9) δ = 169.0, 168.9, 168.9, 166.7, 151.4, 150.6, 150.6, 150.5, 150.3, 150.3, 150.2, 150.2, 150.2, 150.1, 150.1, 149.7, 148.9, 129.8, 128.8, 128.8, 128.5, 128.4, 128.0, 127.7, 126.6, 126.4, 119.0, 115.5, 113.8, 113.7, 113.5, 113.4, 113.0, 112.9, 112.5, 112.5, 112.4, 112.4, 77.3, 71.8, 71.8, 69.9, 69.9, 69.9, 69.6, 68.5, 67.8, 66.0, 57.0, 56.4, 56.0, 55.8, 55.6, 55.5, 55.3, 55.2, 39.5, 37.5, 31.9, 31.7, 29.8, 29.7, 28.6, 28.5, 27.2, 23.2, 22.3, 22.3, 19.5, 14.1; MS (m/z): HRMS (ESI) Calcd. for C_64_H_75_FeN_2_${\text{O}}_{12}^{+}$ ([M + H]^+^): 1119.4671, found: 1119.4669 (Figure S10).

#### P[5]^4^R

Yellow solid, 42.9%, m.p. 107.4-109.2°C; ^1^H NMR (400 MHz, CDCl_3_) δ 7.02–6.76 (m, 10 H, ArH), 5.67 (s, 1 H, NH), 5.26 (s, 1 H, NH), 4.75 (s, 2 H, CH_2_), 4.59 (s, 2 H, ArH), 4.40 (s, 2 H, ArH), 4.24 (d, *J* = 2.4 Hz, 5 H, ArH), 4.05–3.54 (m, 36 H, 24 OCH_3_, 12 CH_2_), 2.72–2.47 (m, 4 H, CH_2_), 1.76 (dd, *J* = 15.2, 8.0 Hz, 2 H, CH_2_), 1.52 (q, *J* = 7.6 Hz, 2 H, CH_2_), 0.96 (t, *J* = 7.6 Hz, 3 H, CH_3_), −0.18 (s, 2 H, CH_2_), −0.90 (s, 1 H, CH_2_), −1.09 (s, 1 H, CH_2_), −1.61 (d, *J* = 23.6 Hz, 2 H, CH_2_), −2.21 (s, 2 H, CH_2_); ^13^C NMR (101 MHz, CDCl_3_) (Figure S13) δ = 169.25, 167.51, 150.91, 150.73, 150.56, 150.45, 150.40, 150.36, 150.32, 150.21, 150.12, 147.21, 129.75, 129.29, 128.75, 128.48, 128.45,128.19, 127.87, 127.82, 127.05, 115.82, 115.08, 114.71, 114.43, 114.00, 112.80, 112.78, 112.73, 112.23, 70.21, 68.88, 68.09, 67.81, 65.85, 56.83, 56.44, 56.29, 56.26, 56.08, 55.48, 55.43, 55.31, 55.10, 39.73, 37.87, 31.95, 30.15, 29.36, 28.89, 28.60, 28.44, 26.37, 24.41, 23.25, 19.57, 14.06; HRMS (ESI) Calcd. for C_66_H_79_FeN_2_${\text{O}}_{12}^{+}$ ([M + H]^+^): 1147.4981, found: 1147.4982 (Figure S14).

#### P[5]^6^R

Yellow solid, 38.9%, m.p. 109.9-112.1°C; ^1^H NMR (400 MHz, CDCl_3_) δ: 6.98–6.70 (m, 10 H, ArH), 5.85 (s, 1 H, NH), 5.18 (s, 1 H, NH), 4.72 (s, 2 H, CH_2_), 4.58 (s, 2 H, ArH), 4.39 (s, 2 H, ArH), 4.24 (s, 5 H, ArH), 4.00–3.59 (m, 36 H, 24 OCH_3_, 12 CH_2_), 3.42 (s, 2 H, CH_2_), 3.29 (s, 2 H, CH_2_), 1.86–1.79 (m, 2 H, CH_2_), 1.60 (q, *J* = 7.6 Hz, 2 H, CH_2_), 1.35 (s, 2 H, CH_2_), 1.03 (t, *J* = 6.3 Hz, 3 H, CH_3_), 0.72 (s, 2 H, CH_2_), −0.17 (s, 2 H, CH_2_), −1.11 (s, 1 H, CH_2_), −1.25 (s, 1 H, CH_2_), −1.50 (s, 2 H, CH_2_), −2.32 (s, 2 H, CH_2_); ^13^C NMR (101 MHz, CDCl_3_) (Figure S17) δ = 169.86, 150.81, 150.52, 150.48, 150.30, 150.20, 149.99, 129.41, 129.05, 128.35, 128.24, 128.09, 127.85, 127.34, 115.04, 114.18, 114.13, 113.70, 112.76, 112.33, 77.34, 70.43, 69.72, 68.19, 68.11, 55.99, 55.69, 55.46, 55.39, 55.29, 55.12, 40.01, 37.99, 32.02, 30.71, 30.11, 29.27, 29.01, 28.89, 28.62, 28.41, 28.27, 27.72, 19.65, 14.14; MS (m/z): HRMS (ESI) Calcd. for C_68_H_83_FeN_2_${\text{O}}_{12}^{+}$ ([M + H]^+^): 1175.5294, found: 1175.5295 (Figure S18).

#### P[5]^8^R

Yellow solid, 25.9%, m.p. 114.6-116.8°C; ^1^H NMR (400 MHz, CDCl_3_) δ 6.95–6.80 (m, 9H, ArH), 6.71 (s, 1H, ArH), 5.23 (s, 1H, NH), 5.02 (s, 1H, NH), 4.68 (t, *J* = 1.9 Hz, 2H, CH_2_), 4.56 (s, 2H, ArH), 4.37 (t, *J* = 2.0 Hz, 2H, ArH), 4.22 (s, 5H, ArH), 3.92–3.63 (m, 36H, 24OCH_3_, 12CH_2_), 3.42 (q, *J* = 7.0 Hz, 2H, CH_2_), 2.41 (s, 2H, CH_2_), 1.88–1.79 (m, 2H, CH_2_), 1.62 (td, *J* = 7.4, 2.6 Hz, 4H, CH_2_), 1.37 (p, *J* = 7.7 Hz, 2H, CH_2_), 1.20 (t, *J* = 7.9 Hz, 2H, CH_2_), 1.04 (t, *J* = 7.4 Hz, 3H, CH_3_), 0.80 (s, 2H, CH_2_), −0.05 (s, 2H, CH_2_), −1.34 (s, 4H, CH_2_), −2.38 (s, 2H, CH_2_); ^13^C NMR (101 MHz, CDCl_3_) (Figure S21) δ = 170.09, 167.19, 150.80, 150.37, 150.24, 150.12, 150.06, 149.95, 146.97, 129.40, 129.01, 128.32, 128.20, 128.11, 127.94, 127.84, 127.83, 127.08, 114.73, 113.92, 113.58, 113.25, 112.73, 112.42, 76.31, 70.43, 69.72, 68.00, 67.82, 55.48, 55.45, 55.36, 55.32, 55.13, 39.72, 38.02, 32.05, 30.96, 30.66, 30.60, 30.21, 29.64, 29.26, 28.83, 28.76, 28.64, 28.22, 27.94, 19.65, 14.17; IR (KBr) υ: 3410, 2932, 2854, 1681, 1499, 1465, 1399, 1295, 1213, 1104, 1047, 929, 879, 855, 774, 704, 647cm^−1^; MS (m/z): HRMS (ESI) Calcd. for C_70_H_87_FeN_2_${\text{O}}_{12}^{+}$ ([M+H]^+^): 1203.5602, found: 1203.5508 (Figure S22).

#### Mono-ferrocene Modified Pillar[5]arene P[5]^0^R

Yellow solid, 78.6%, m.p. 104.4–106.2°C; ^1^H NMR (400 MHz, CDCl_3_) (Figure S1) δ: 6.78–6.82 (m, 4 H, ArH), 6.76 (d, *J* = 2.7 Hz, 2 H, ArH), 6.70 (s, 1 H, ArH), 6.65 (s, 1 H, ArH), 4.68 (t, *J* = 1.9 Hz, 2 H, ArH), 4.37 (s, 2 H, CH_2_), 4.32 (t, *J* = 1.9 Hz, 2 H, ArH), 4.19 (s, 5 H, ArH), 3.88 (t, *J* = 6.4 Hz, 2 H, CH_2_), 3.85–3.62 (m, 28 H, 24 OCH_3_, 4 CH_2_), 3.60 (s, 3 H, CH_2_), 3.56 (s, 3 H, CH_2_), 3.24 (s, 2 H, CH_2_), 3.11 (s, 2 H, CH_2_), 1.72–1.82 (m, 2 H, CH_2_), 1.53 (h, *J* = 7.4 Hz, 2 H, CH_2_), 0.97 (t, *J* = 7.4 Hz, 3 H, CH_3_); ^13^C NMR (101 MHz, CDCl_3_) (Figure S2) δ = 170.70, 151.19, 150.87, 150.82, 150.77, 150.76, 150.69, 150.66, 148.15, 129.28,129.23, 128.62, 128.46, 128.36, 128.08, 127.84, 127.72, 115.41, 115.34, 114.37, 114.31, 114.06, 114.03, 113.97, 113.90, 113.79, 76.13, 70.33, 69.70, 68.37, 68.24, 67.67, 56.68, 56.17, 56.06, 55.91, 55.87, 55.80, 55.77, 41.21, 38.87, 31.80, 30.21, 29.70, 29.64, 28.76, 19.50, 13.96; MS (m/z): HRMS (ESI) Calcd. for C_62_H_71_FeN_2_${\text{O}}_{12}^{+}$ ([M + H]^+^): 1091.4357, found: 1091.4356 (Figure S3).

#### P[5]^1^R

Yellow solid, 71.9 %, m.p. 105.6-107.3°C;^1^H NMR (400 MHz, CDCl_3_) (Figure S5) δ: 6.75–6.98 (m, 10H, ArH), 6.60 (s, 2 H, NH), 4.77 (t, *J* = 2.0 Hz, 2 H, ArH), 4.39 (s, 2 H, CH_2_), 4.34 (t, *J* = 1.9 Hz, 2 H, ArH), 4.20 (s, 5 H, ArH), 3.46–3.97 (m, 36 H, 24 OCH_3_, 12 CH_2_), 1.81 (p, *J* = 6.9 Hz, 2H, CH_2_), 1.68 (s, 2 H, CH_2_), 1.56 (q, *J* = 7.5 Hz, CH_2_), 1.01 (t, *J* = 7.4 Hz, 3 H, CH_3_); ^13^C NMR (101 MHz,CDCl_3_) (Figure S6) δ = 150.7, 150.6, 150.4, 148.6, 128.8, 128.3, 128.1, 128.1, 114.6, 114.6, 114.5, 114.4, 113.7, 113.4, 113.3, 113.2, 70.0, 70.0, 69.6, 68.2, 66.9, 56.2, 56.2, 56.2, 56.2, 55.9, 55.9, 55.7, 55.5, 39.4, 35.7, 34.8, 31.9, 29.7, 29.4, 19.5, 14.1; MS (m/z): HRMS (ESI) Calcd. for C_63_H_73_FeN_2_${\text{O}}_{12}^{+}$ ([M + H]^+^): 1105.4512, found: 1105.4513 (Figure S7).

### Synthesis of Monomer M^3^

**Scheme 2:**
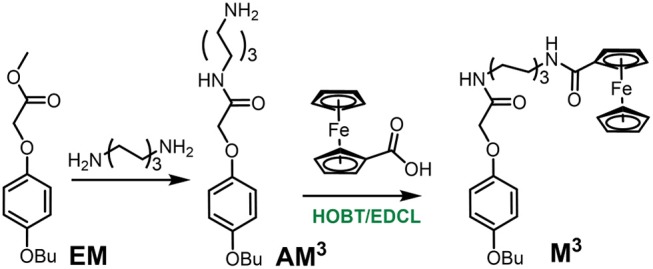
Synthetic route to monomer **M**^**3**^.

**AM**^**3**^ was obtained from a previous report. Then the monomer **M**^**3**^ was synthesized from **AM**^**3**^ (Figure S23) and ferrocene-carboxylic acid with BOBT and EDCL as the catalyst (Scheme 2). **AM**^**3**^ (0.08g, 0.25 mmol), ferrocenecarboxylic acid (0.057 g, 0.25 mmol), HOBT (0.054 g, 0.40 mmol), and EDCL (0.076, 0.40 mmol) were stirred in 15 mL dry CHCl_3_ over night at room temperature. The reaction solvent was evaporated and the residue was purified by flash column chromatography on silica gel (CH_2_Cl_2_/CH_3_OH, *v*/*v* 25:1) to give **M**^**3**^ as a yellow solid (0.031 g). ^1^H NMR (400 MHz, CDCl_3_) (Figure S24) δ 6.84 (s, 4 H, ArH), 6.65 (s, 1 H, NH), 5.87 (s, 1 H, NH), 4.68 (s, 2 H, CH_2_), 4.44 (s, 2 H, ArH), 4.33 (s, 2 H, ArH), 4.19 (s, 5 H, ArH), 3.91 (t, *J* = 5.8 Hz, 2 H, CH_2_), 3.36 (d, *J* = 6.2 Hz, 4 H, CH_2_), 1.80–1.68 (m, 4 H, CH_2_), 1.58 (d, *J* = 5.9 Hz, 4 H, CH_2_), 1.48 (dd, *J* = 14.6, 7.1 Hz, 2 H, CH_2_), 1.39 (s, 2 H, CH_2_), 0.97 (t, *J* = 6.9 Hz, 3 H, CH_3_).

## Materials

All reactions were performed in atmosphere unless noted. All reagents were commercially available and used as supplied without further purification. NMR spectra were collected on either a Bruker AVIII-400 MHz spectrometer or a Bruker AV-600 MHz spectrometer with internal standard tetramethylsilane (TMS) and signals as internal references, and the chemical shifts (δ) were expressed in ppm. High-resolution Mass (ESI) spectra were obtained with a Bruker Micro-TOF spectrometer. X-ray data were collected on a Bruker Smart APEX-2 CCD diffractometer.

## Results and Disscussion

### ^1^H NMR Investigation

**Figure 1:**
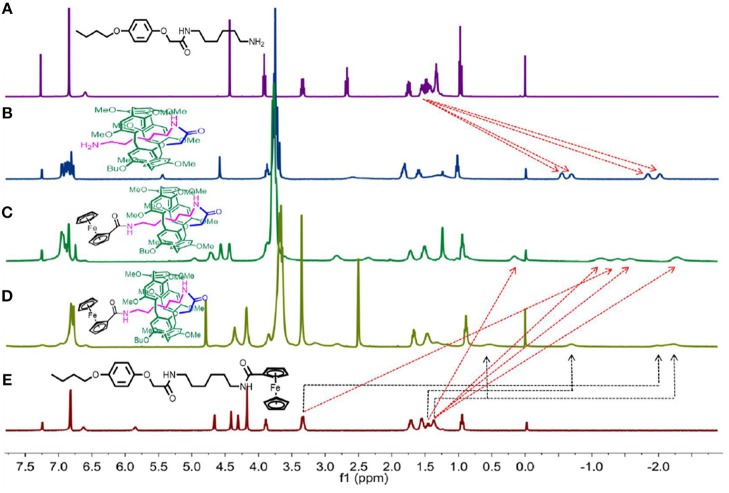
^1^H NMR spectra (400 MHz, 298K) of: **(A) AM**^**3**^ in CDCl_3_; **(B) P[5]**^**4**^**PR** in CDCl_3_; **(C) P[5]**^**4**^**R** in CDCl_3_; **(D) P[5]**^**4**^**R** in DMSO-*d*_6_; **(E) M**^**3**^ in CDCl_3_.

The ^1^H NMR spectra of **AM**^**3**^ and **P[5]**^**4**^**PR** were taken into consideration first. As shown in Figure 1B, the chemical shift of four groups of peaks shift below 0 ppm field, indicating that the alkyl chain penetrated into the cavity of pillar[5]arene to form either pseudo[1] rotaxane or [c2]daisy chain (Du et al., 2017). Then **P[5]**^**4**^**R** was prepared from **P[5]**^**4**^**PR** reacted with ferrocenecarboxylic acid as the stopper. ^1^H NMR spectra of monomer **M**^**3**^ and [1] rotaxane **P[5]**^**4**^**R** in CDCl_3_ at 293 K are shown in Figure 1 (spectra c and e). Compared with **M**^**3**^, we found that the signals of protons on the alkyl chain attaching onto the pillar[5]arene platform shifted upfield obviously due to the shielding effect (Figure 1C). Then we used a polar solvent, DMSO-*d*_6_, for ^1^H NMR investigations to confirm the formation of [1] rotaxane. In DMSO-*d*_6_, we also found that the signals of protons on the alkyl chains upfield were below 0 ppm due to the shielding effect (Figure 1D), which indicated the formation of a mechanically interlocked structure (Dong et al., 2014). The ^1^H NMR of **P[5]**^**2**^**R**, **P[5]**^**4**^**R**, **P[5]**^**6**^**R**, **P[5]**^**8**^**R** all showed several groups of protons on the alkyl chains upfield obviously (Figures S8, S12, S16, S20), and the formation of [1] rotaxanes was also confirmed. However, the ^1^H NMR of **P[5]**^**0**^**R** and **P[5]**^**1**^**R** showed no signal below 0 ppm, indicating the side-chain stayed outside of the cavity of the pillar[5]arene platform (Figures S1, S5). The reason for this phenomenon is due to the relatively short length of the axle (only two or three CH_2_ groups) of **P[5]**^**0**^**R**, and **P[5]**^**1**^**R**, which was not able to allow the large ferrocene group to connect it from the cavity. Thus, the amino-group of the side-chain of **P[5]**^**0**^**PR** (or **P[5]**^**1**^**PR**) stayed outside of the cavity and was then reacted with ferrocene-carboxylic acid to obtain free form **P[5]**^**0**^**R** (or **P[5]**^**1**^**R**). Furthermore, the temperature-dependent ^1^H NMR of **P[5]4R** showed that the peaks became broad as the temperature increased, indicating the chain in the cavity (Figures S15, S19, S26).

### 2D NOESY Studies

**Figure 2:**
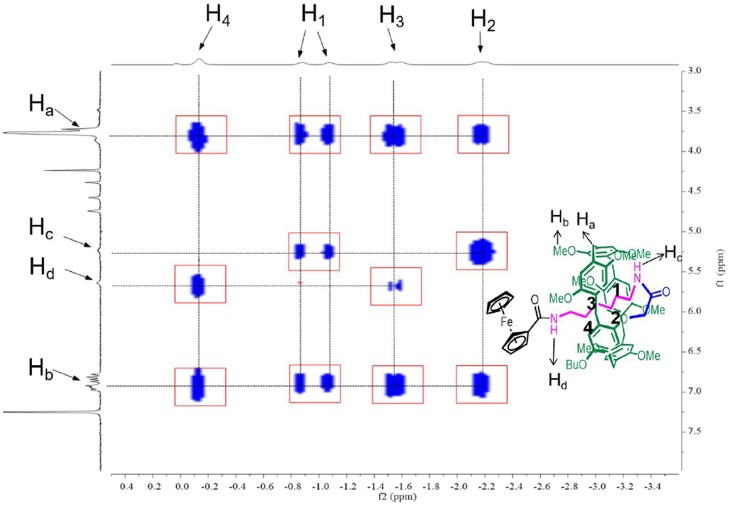
Partial 2D NOESY spectrum of a choroform-d solution of **P[5]**^**4**^**R** at 298K.

The formation of [1]rotaxane was then confirmed by 2D Nuclear Overhauser Effect Spectroscopy (NOESY). Here we also take **P[5]**^**4**^**R** as the model compound. As shown in Figure 2, the hydrogens of the alkyl chain on **P[5]**^**4**^**R** were close to the pillar[5]arene platform because H_1−4_ showed strong correlation with H_a_ and H_b_, indicating that the alkyl chain was in close proximity to the cavity. The -NH- group H_c_ is close to H_1−2_ while H_d_ is close to H_3−4_. Furthermore, ArH-3 from the ferrocene group showed space correction to the hydrogen–OCH_3_ and -OCH_2_- on the pillar[5]arene platform (Data Sheets 1–4).

### Single Crystal Structures

**Figure 3:**
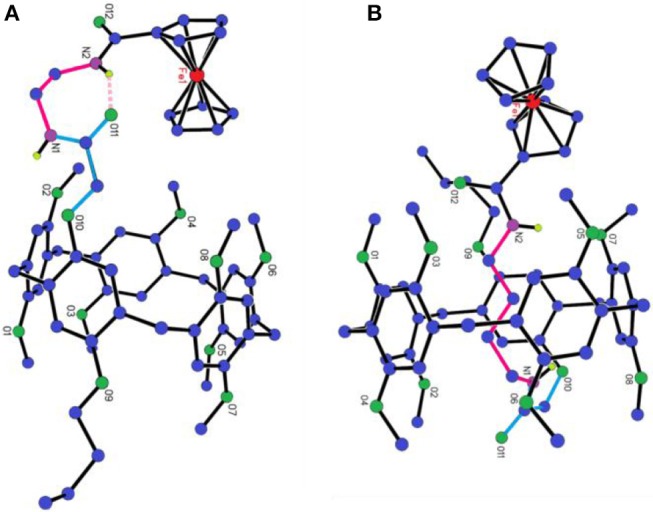
X-ray single-crystal structure of: **(A) P[5]**^**0**^**R**; **(B) P[5]**^**2**^**R**. Color code: C, blue; O, green; Fe, red; N, purple.

The direct evidence for the formation of [1] rotaxanes only when the length of axle longer than three CH_2_ groups is from single crystal investigation. As shown in Figure 3A and Figure S4, the whole side chain of **P[5]**^**0**^**R** stayed outside of the cavity of pillar[5]arene. It should be pointed that we observed hydrogen bonding between the hydrogen atom of the amine group and the oxygen atom of carbonyl group (Figure 3A, pink dash line). However, for **P[5]**^**2**^**R**, we can clearly see that the alkyl chain penetrated into the cavity of pillar[5]arene to form a [1] rotaxane (Figure 3B and Figure S11). The C-H…π interactions and C-H…O interactions were the driving forces for the formation of [1] rotaxane.

### Cyclic Voltammetry Investigation

**Figure 4:**
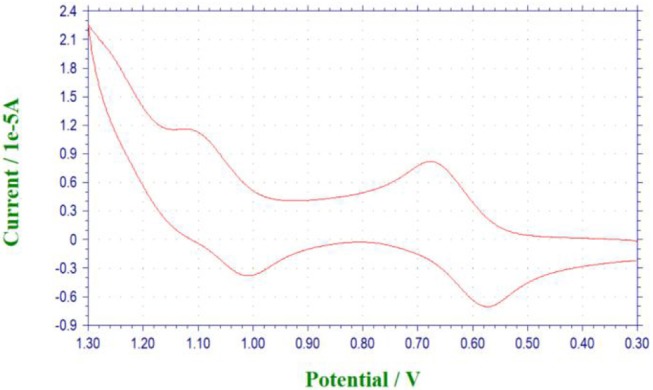
Cyclic voltammogram (scan rate = 100 mV s^−1^) of the **P[5]**^**4**^**R** (5.00 × 10^−4^ M) in CH_2_Cl_2_.

With the [1]rotaxanes in hand, we then investigated their reversible redox property by electrochemistry methods. Take **P[5]**^**4**^**R** as an example, in cyclic voltammetry (CV) experiment (Figure 4), the cyclic voltammogram was quasi-reversible with nearly equal *i*_pa_ and *i*_pc_, in which the potential difference Δ*E*_p_ was around 0.090 V. Compared with ferrocene, **P[5]**^**4**^**R** has a larger half wave potential (E_1/2_ = 612 mV). Further study showed that the free state **P[5]**^**0**^**R** has the similar redox property with **P[5]**^**4**^**R** due to the same ferrocene unit (Figure S25).

## Conclusions

In this paper, we synthesized a series of amide-linked pillar[5]arene-based [1]rotaxanes with ferrocene unit as the stopper. Under the catalysis of HOBT/EDCL, the mono-amido-functionalized pillar[5]arenes were amidated with ferrocene carboxylic acid, to constructed ferrocene-based [1]rotaxanes, respectively. The structure of the [1]rotaxanes were characterized by ^1^H NMR, ^13^C NMR, 2D NMR, mass spectroscopy and single-crystal X-ray structural determination. In the formation of [1]rotaxane, the key role is the length of the alkyl chain in this process, and only when the number of C on the alkyl chain is larger than three can the formation of [1]rotaxane occur. In addition, due to the ferrocene units, the pillar[5]arene-based [1]rotaxanes display electrochemically reversible properties. Based on this nature, we hope these pillar[5]arene-based [1]rotaxanes can be applied in battery devices in future.

## Data Availability

The raw data supporting the conclusions of this manuscript will be made available by the authors, without undue reservation, to any qualified researcher.

## Author Contributions

RZ prepared all the pillar[5]arene-based [1]rotaxanes. CW and RL prepared the monomer M^3^. TC and CY analyzed the data. YY analyzed the data and wrote the paper.

### Conflict of Interest Statement

The authors declare that the research was conducted in the absence of any commercial or financial relationships that could be construed as a potential conflict of interest.
