# Supplementary material for: Pillar[5]arene Based [1]rotaxane Systems With Redox-Responsive Host-Guest Property: Design, Synthesis and the Key Role of Chain Length
Source: Front Chem. 2019 Jul 23;7:508. doi: 10.3389/fchem.2019.00508 (PMC6663970; doi:10.3389/fchem.2019.00508)
Supplement: Supplementary file 1 [file Table_1.DOC]

Pillar[5]arene based [1]rotaxne systems with redox-responsive host-guest property: design, synthesis and the key role of chain length

[*Runmiao Zhang*](http://loop.frontiersin.org/people/265469/overview)*1, 2 , Chenwei Wang^1^, Renhua Long^1^, Tingting Chen^1^*, Chaoguo Yan^2^*, and Yong Yao^1^**

*1 College of Chemistry and Chemical Engineer, Nantong University, Nantong, Jiangsu, 226019, P.R. China, 2 College of Chemistry and Chemical Engineer, Yangzhou University, Yangzhou, Jiangsu, 225000, P.R. China*

Figure S1. ^1^H NMR spectra of **P[5]^0^R** in CDCl_3_ at 400 MHz (298 K).

Figure S2. ^13^C NMR spectra of **P[5]^0^R** in CDCl_3_ at 101 MHz (298 K).

Figure S3. Mass spectra of **P[5]^0^R**.

Figure S4. Single crystal structure of **P[5]^0^R**.

CCDC number: 1937458. Crystallographic data: block, yellow, 0.24 × 0.22 × 0.20 mm^3^, C_62_H_70_FeN_2_O_12_, *FW* 1091.05, monoclinic, space group *P* bca, *a* = 20.7599(9), *b* = 14.7083(7), *c* = 37.9438(15) Å, *α* = 90°, *β* = 90°, *γ* = 90°, *V =*11585.9(9) Å^3^, *Z* = 8, *D_c_* =1.251 g∙cm^-3^, *T* =273 (2) K, *μ*=0.323mm^-1^, 93901 measured reflections, 11383 independent reflections, 741 parameters, 62 restraints, *F*(000) = 4624, *R*(int) = 0.0982, *R*_1_ = 0.1109, *wR*_1_ = 0.1292 (all data), *R*_2_ = 0.0545, *wR*_2_ = 0.1094[*I*＞2σ(*I*)], max.residual density 0.482 e•Å^-3^, and goodness-of-fit (*F^2^*) = 1.012.

Figure S5. ^1^H NMR spectra of **P[5]^1^R** in CDCl_3_ at 400 MHz.

Figure S6. ^13^C NMR spectra of **P[5]^1^R** in CDCl_3_ at 101 MHz.

Figure S7. Mass spectra of **P[5]^0^R**.

Figure S8. ^1^H NMR spectra of **P[5]^2^R** in CDCl_3_ at 400 MHz.

Figure S9. ^13^C NMR spectra of **P[5]^2^R** in CDCl_3_ at 101 MHz.

Figure S10. Mass spectra of **P[5]^2^R**.

Figure S11. Single crystal structure of **P[5]^2^R**.

CCDC number: 1937459. Crystallographic data: block, yellow, 0.26 × 0.22 × 0.18 mm^3^, C_66_H_80_FeN_2_O_13_, *FW* 1165.17, monoclinic, space group *P* -1, *a* = 12.032(4), *b* = 13.719(5), *c* = 19.531(7) Å, *α* = 90.395(10)°, *β* = 91.990(10)°, *γ* = 111.692(10)°, *V =*2993.2(18) Å^3^, *Z* = 2, *D_c_* =1.293 g∙cm^-3^, *T* =296 (2) K, *μ*=0.318mm^-1^, 25151 measured reflections, 11236 independent reflections, 764 parameters, 46 restraints, *F*(000) = 1240, *R*(int) = 0.0909, *R*_1_ = 0.1895, *wR*_1_ = 0.2251 (all data), *R*_2_ = 0.0709, *wR*_2_ = 0.1718[*I*＞2σ(*I*)], max.residual density 0.675 e•Å^-3^, and goodness-of-fit (*F^2^*) = 0.980.

Figure S12. ^1^H NMR spectra of **P[5]^4^R** in CDCl_3_ at 400 MHz.

Figure S13. ^13^C NMR spectra of **P[5]^4^R** in CDCl_3_ at 101 MHz.

Figure S14. Mass spectra of **P[5]^4^R**.

Figure S15. 2D NOESY spectrum of **P[5]^4^R** in CDCl_3_ at 400 MHz.

Figure S16. ^1^H NMR spectra of **P[5]^6^R** in CDCl_3_ at 400 MHz.

Figure S17. ^13^C NMR spectra of **P[5]^6^R** in CDCl_3_ at 101 MHz.

Figure S18. Mass spectra of **P[5]^6^R**.

Figure S19. 2D NOESY spectrum of **P[5]^6^R** in CDCl_3_ at 400 MHz.

Figure S20. ^1^H NMR spectra of **P[5]^8^R** in CDCl_3_ at 400 MHz.

Figure S21. ^13^C NMR spectra of **P[5]^8^R** in CDCl_3_ at 101 MHz.

Figure S22. Mass spectra of **P[5]^8^R**.


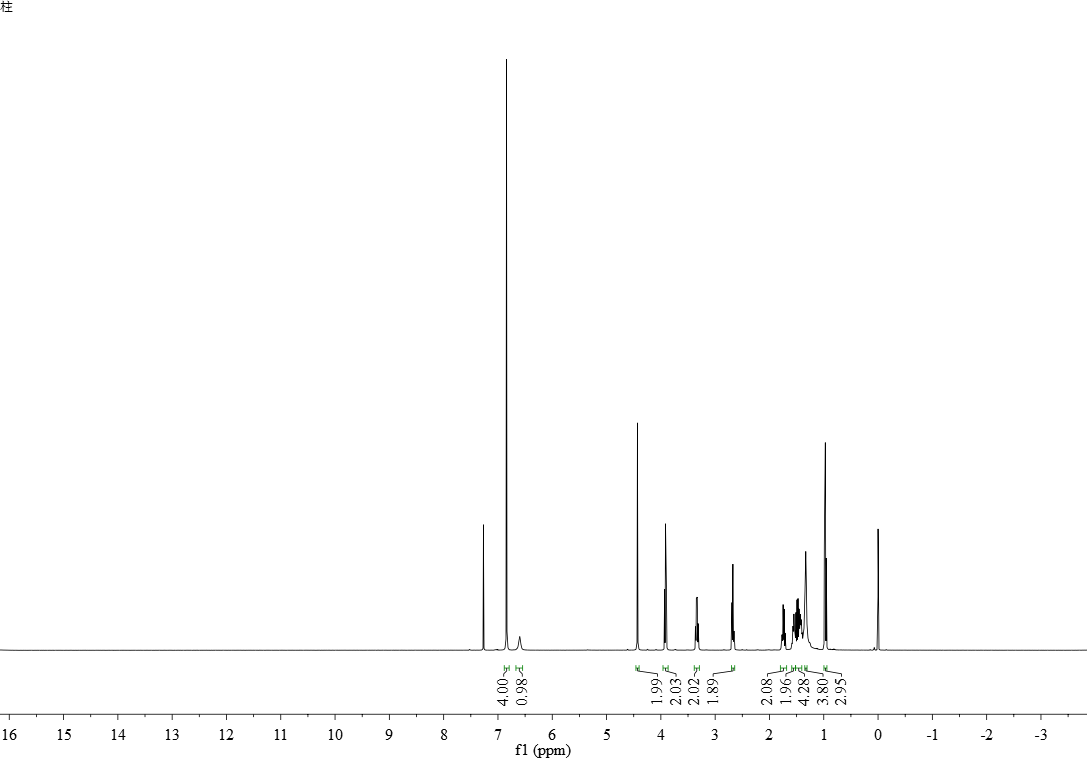


Figure S23. ^1^H NMR spectra of **AM^3^** in CDCl_3_ at 400 MHz.


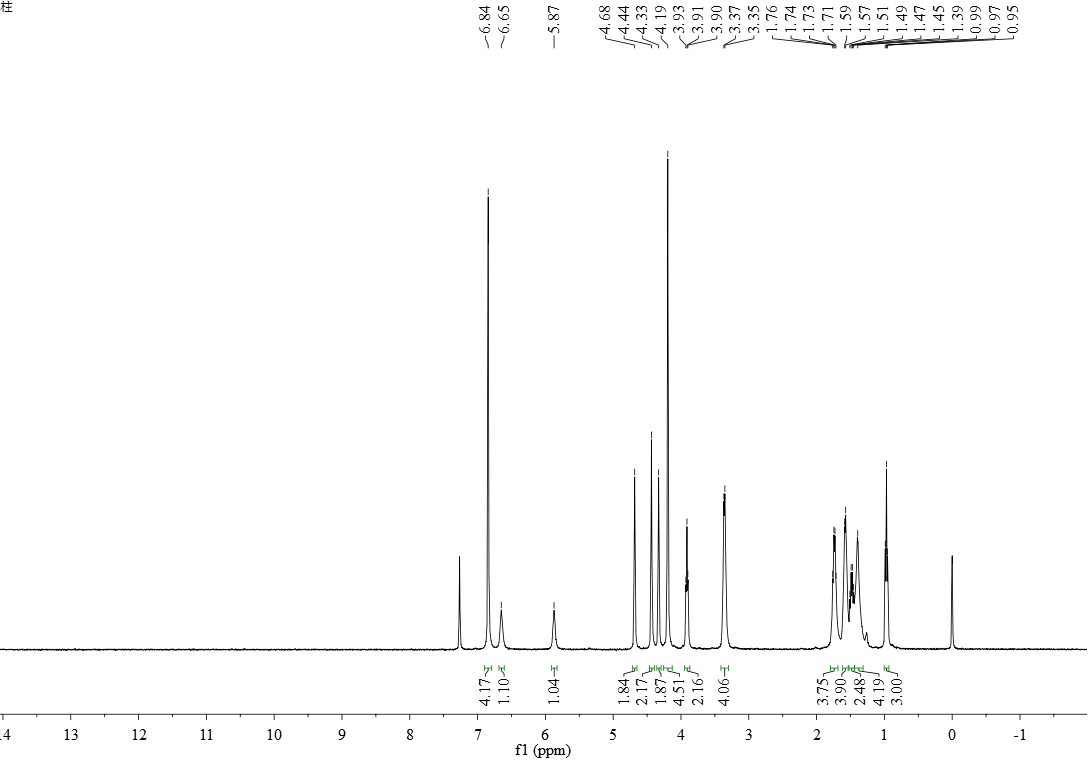


Figure S24. ^1^H NMR spectra of **M^3^** in CDCl_3_ at 400 MHz.


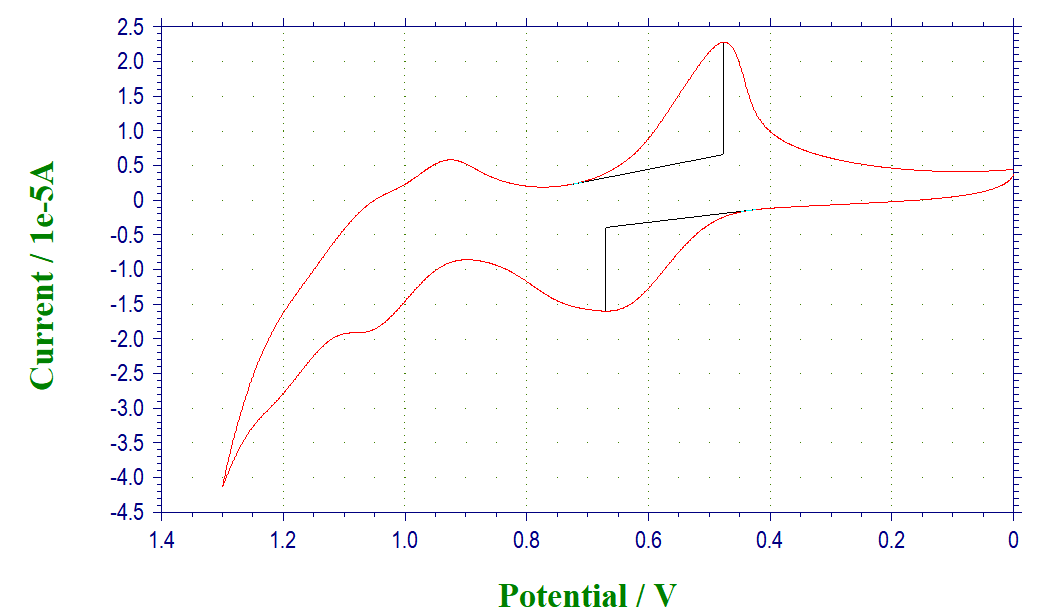


Figure S25. The cyclic voltammetric curves of **P[5]^0^R**.

Figure S26. ^1^H NMR spectra of **P[5]^4^R** in CDCl_3_ at 600MHz in the range of 20-50℃.
