# Supplementary material for: Pillar[5]arene Based [1]rotaxane Systems With Redox-Responsive Host-Guest Property: Design, Synthesis and the Key Role of Chain Length
Source: Front Chem. 2019 Jul 23;7:508. doi: 10.3389/fchem.2019.00508 (PMC6663970; doi:10.3389/fchem.2019.00508)

checkCIF/PLATON report

Structure factors have been supplied for datablock(s) zrm190127_a_sq

THIS REPORT IS FOR GUIDANCE ONLY. IF USED AS PART OF A REVIEW PROCEDURE FOR PUBLICATION, IT SHOULD NOT REPLACE THE EXPERTISE OF AN EXPERIENCED CRYSTALLOGRAPHIC REFEREE.

No syntax errors found. CIF dictionary Interpreting this report

Datablock: zrm190127_a_sq

Bond precision: C-C = 0.0042 A Wavelength=0.71073

Cell:	a=20.7599(9)	b=14.7083(7)	c=37.9438(15)
	alpha=90	beta=90	gamma=90
Temperature:	273 K		

	Calculated	Reported
Volume	11585.9(9)	11585.9(9)
Space group	P b c a	P b c a
Hall group	-P 2ac 2ab	-P 2ac 2ab
Moiety formula	C62 H70 Fe N2 O12 [+ solvent]	C62 H70 Fe N2 O12
Sum formula	C62 H70 Fe N2 O12 [+ solvent]	C62 H70 Fe N2 O12
Mr	1091.05	1091.05
Dx,g cm-3	1.251	1.251
Z	8	8
Mu (mm-1)	0.323	0.323
F000	4624.0	4624.0
F000’	4628.88	
h,k,lmax	25,18,46	25,18,46
Nref	11392	11383
Tmin,Tmax	0.925,0.937	0.925,0.937
Tmin’	0.925	

Correction method= # Reported T Limits: Tmin=0.925 Tmax=0.937
AbsCorr = MULTI-SCAN

Data completeness= 0.999 Theta(max)= 25.999

R(reflections)= 0.0545(7039) wR2(reflections)= 0.1292(11383)

S = 1.012 Npar= 741

The following ALERTS were generated. Each ALERT has the format
test-name_ALERT_alert-type_alert-level.
Click on the hyperlinks for more details of the test.

Alert level C

```
PLAT220_ALERT_2_C Non-Solvent  Resd 1  C   Ueq(max)/Ueq(min) Range        4.0 Ratio
PLAT222_ALERT_3_C Non-Solv.  Resd 1  H   Uiso(max)/Uiso(min) Range        4.6 Ratio
PLAT411_ALERT_2_C Short Inter H...H Contact  H58      ..H44C     .       2.05 Ang.
                                                x,1+y,z  =      1_565 Check
PLAT910_ALERT_3_C Missing # of FCF Reflection(s) Below Theta(Min).          6 Note
PLAT911_ALERT_3_C Missing FCF Refl Between Thmin & STh/L=    0.600          3 Report
```

Alert level G

```
PLAT003_ALERT_2_G Number of Uiso or Uij Restrained non-H Atoms ...          9 Report
PLAT007_ALERT_5_G Number of Unrefined Donor-H Atoms ..............          2 Report
PLAT066_ALERT_1_G Predicted and Reported Tmin&Tmax Range Identical          ? Check
PLAT083_ALERT_2_G SHELXL Second Parameter in WGHT  Unusually Large       9.61 Why ?
PLAT177_ALERT_4_G The CIF-Embedded .res File Contains DELU Records          3 Report
PLAT178_ALERT_4_G The CIF-Embedded .res File Contains SIMU Records          3 Report
PLAT199_ALERT_1_G Reported _cell_measurement_temperature ..... (K)        273 Check
PLAT200_ALERT_1_G Reported   _diffrn_ambient_temperature ..... (K)        273 Check
PLAT301_ALERT_3_G Main Residue  Disorder ..............(Resd  1  )         5% Note
PLAT432_ALERT_2_G Short Inter X...Y Contact  C17      ..C22              3.19 Ang.
                                         1-x,-1/2+y,1/2-z  =      4_645 Check
PLAT605_ALERT_4_G Largest Solvent Accessible VOID in the Structure        128 A**3
PLAT794_ALERT_5_G Tentative Bond Valency for Fe1       (II)      .       2.16 Info
PLAT860_ALERT_3_G Number of Least-Squares Restraints .............         62 Note
PLAT869_ALERT_4_G ALERTS Related to the Use of SQUEEZE Suppressed           ! Info
PLAT883_ALERT_1_G No Info/Value for _atom_sites_solution_primary .     Please Do !
PLAT933_ALERT_2_G Number of OMIT Records in Embedded .res File ...          5 Note
PLAT978_ALERT_2_G Number C-C Bonds with Positive Residual Density.          1 Info
```

```
   0 ALERT level A = Most likely a serious problem - resolve or explain
   0 ALERT level B = A potentially serious problem, consider carefully
   5 ALERT level C = Check. Ensure it is not caused by an omission or oversight
  17 ALERT level G = General information/check it is not something unexpected

   4 ALERT type 1 CIF construction/syntax error, inconsistent or missing data
   7 ALERT type 2 Indicator that the structure model may be wrong or deficient
   5 ALERT type 3 Indicator that the structure quality may be low
   4 ALERT type 4 Improvement, methodology, query or suggestion
   2 ALERT type 5 Informative message, check
```

It is advisable to attempt to resolve as many as possible of the alerts in all categories. Often the minor alerts point to easily fixed oversights, errors and omissions in your CIF or refinement strategy, so attention to these fine details can be worthwhile. In order to resolve some of the more serious problems it may be necessary to carry out additional measurements or structure refinements. However, the purpose of your study may justify the reported deviations and the more serious of these should normally be commented upon in the discussion or experimental section of a paper or in the "special_details" fields of the CIF. checkCIF was carefully designed to identify outliers and unusual parameters, but every test has its limitations and alerts that are not important in a particular case may appear. Conversely, the absence of alerts does not guarantee there are no aspects of the results needing attention. It is up to the individual to critically assess their own results and, if necessary, seek expert advice.

Publication of your CIF in IUCr journals

A basic structural check has been run on your CIF. These basic checks will be run on all CIFs submitted for publication in IUCr journals (*Acta Crystallographica*, *Journal of Applied Crystallography*, *Journal of Synchrotron Radiation*); however, if you intend to submit to *Acta Crystallographica Section C* or *E* or *IUCrData*, you should make sure that full publication checks are run on the final version of your CIF prior to submission.

Publication of your CIF in other journals

Please refer to the *Notes for Authors* of the relevant journal for any special instructions relating to CIF submission.

PLATON version of 03/05/2019; check.def file version of 29/04/2019

Datablock zrm190127_a_sq - ellipsoid plot

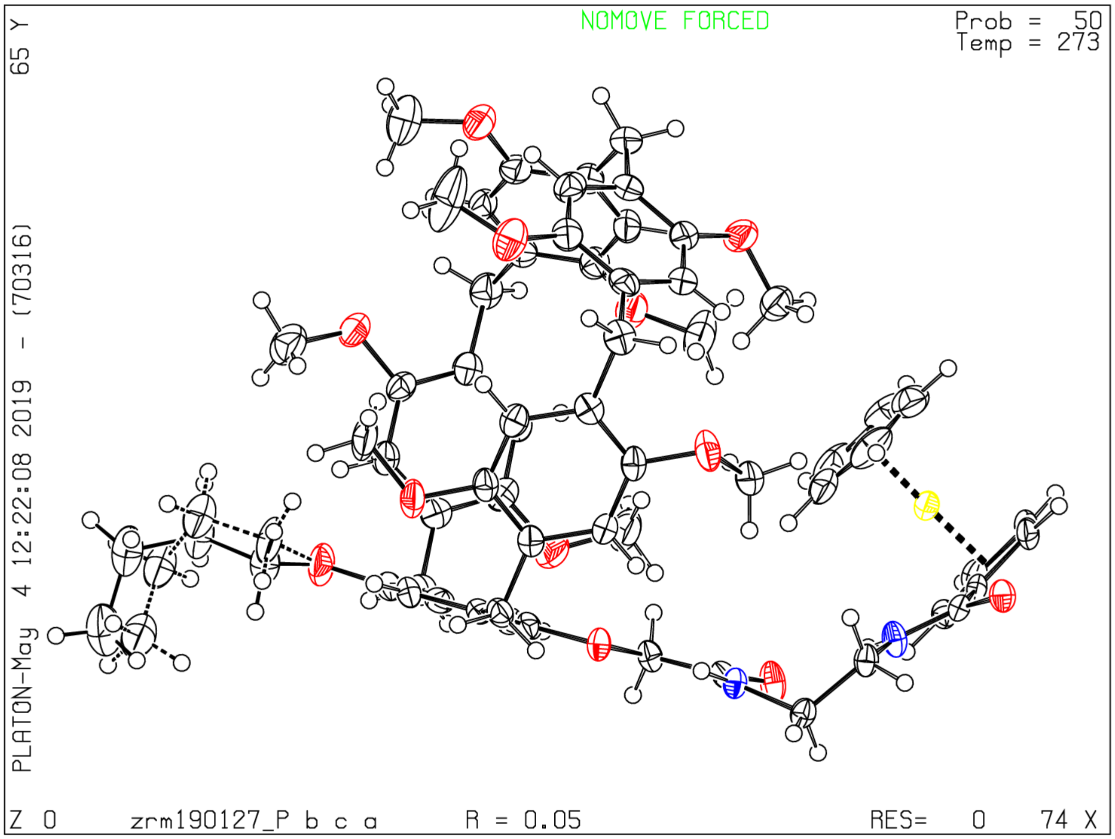

Supplement: Supplementary file 2 [file Data_Sheet_1.PDF]
