# Supplementary material for: Pillar[5]arene Based [1]rotaxane Systems With Redox-Responsive Host-Guest Property: Design, Synthesis and the Key Role of Chain Length
Source: Front Chem. 2019 Jul 23;7:508. doi: 10.3389/fchem.2019.00508 (PMC6663970; doi:10.3389/fchem.2019.00508)

checkCIF/PLATON report

Structure factors have been supplied for datablock(s) ZRM180602

THIS REPORT IS FOR GUIDANCE ONLY. IF USED AS PART OF A REVIEW PROCEDURE FOR PUBLICATION, IT SHOULD NOT REPLACE THE EXPERTISE OF AN EXPERIENCED CRYSTALLOGRAPHIC REFEREE.

No syntax errors found.   CIF dictionary   Interpreting this report

Datablock: ZRM180602

```
Bond precision:  C-C = 0.0069 A          Wavelength=0.71073

Cell:           a=12.032(4)        b=13.719(5)     c=19.531(7)
                alpha=90.395(10)   beta=91.99(1)   gamma=111.692(10)
Temperature:    296 K
```

	Calculated	Reported
Volume	2993.1(18)	2993.2(18)
Space group	P -1	P -1
Hall group	-P 1	-P 1
Moiety formula	C64 H74 Fe N2 O12, C2 H6 O	C64 H74 Fe N2 O12, C2 H6 O
Sum formula	C66 H80 Fe N2 O13	C66 H80 Fe N2 O13
Mr	1165.17	1165.17
Dx,g cm-3	1.293	1.293
Z	2	2
Mu (mm-1)	0.318	0.318
F000	1240.0	1240.0
F000’	1241.26	
h,k,lmax	14,16,24	14,16,24
Nref	11762	11236
Tmin,Tmax	0.921,0.944	0.921,0.944
Tmin’	0.921	

```
Correction method= # Reported T Limits: Tmin=0.921 Tmax=0.944
AbsCorr = MULTI-SCAN

Data completeness= 0.955          Theta(max)= 26.000

R(reflections)= 0.0709( 5443)     wR2(reflections)= 0.2251( 11236)

S = 0.980                  Npar= 764
```

The following ALERTS were generated. Each ALERT has the format
test-name_ALERT_alert-type_alert-level.
Click on the hyperlinks for more details of the test.

Alert level C

```
PLAT026_ALERT_3_C Ratio Observed / Unique Reflections (too) Low ..        48% Check
PLAT241_ALERT_2_C High   'MainMol' Ueq as Compared to Neighbors of        C53 Check
PLAT341_ALERT_3_C Low Bond Precision on  C-C Bonds ...............    0.00691 Ang.
PLAT420_ALERT_2_C D-H Without Acceptor       N2       --H2       .     Please Check
PLAT906_ALERT_3_C Large K Value in the Analysis of Variance ......      4.708 Check
PLAT910_ALERT_3_C Missing # of FCF Reflection(s) Below Theta(Min).          8 Note
PLAT911_ALERT_3_C Missing FCF Refl Between Thmin & STh/L=    0.600        493 Report
```

Alert level G

```
PLAT003_ALERT_2_G Number of Uiso or Uij Restrained non-H Atoms ...          8 Report
PLAT007_ALERT_5_G Number of Unrefined Donor-H Atoms ..............          3 Report
PLAT066_ALERT_1_G Predicted and Reported Tmin&Tmax Range Identical          ? Check
PLAT072_ALERT_2_G SHELXL First  Parameter in WGHT  Unusually Large       0.12 Report
PLAT154_ALERT_1_G The s.u.'s on the Cell Angles are Equal ..(Note)       0.01 Degree
PLAT171_ALERT_4_G The CIF-Embedded .res File Contains EADP Records          4 Report
PLAT177_ALERT_4_G The CIF-Embedded .res File Contains DELU Records          2 Report
PLAT178_ALERT_4_G The CIF-Embedded .res File Contains SIMU Records          2 Report
PLAT230_ALERT_2_G Hirshfeld Test Diff for    O9       --C44      .        6.8 s.u.
PLAT230_ALERT_2_G Hirshfeld Test Diff for    O9       --C44'     .        8.5 s.u.
PLAT301_ALERT_3_G Main Residue  Disorder ..............(Resd  1  )         5% Note
PLAT720_ALERT_4_G Number of Unusual/Non-Standard Labels ..........          5 Note
PLAT794_ALERT_5_G Tentative Bond Valency for Fe1       (II)      .       2.10 Info
PLAT860_ALERT_3_G Number of Least-Squares Restraints .............         46 Note
PLAT883_ALERT_1_G No Info/Value for _atom_sites_solution_primary .     Please Do !
PLAT912_ALERT_4_G Missing # of FCF Reflections Above STh/L=  0.600         29 Note
PLAT933_ALERT_2_G Number of OMIT Records in Embedded .res File ...          4 Note
PLAT978_ALERT_2_G Number C-C Bonds with Positive Residual Density.          2 Info
```

---

 0 **ALERT level A** = Most likely a serious problem - resolve or explain
 0 **ALERT level B** = A potentially serious problem, consider carefully
 7 **ALERT level C** = Check. Ensure it is not caused by an omission or oversight
18 **ALERT level G** = General information/check it is not something unexpected

 3 ALERT type 1 CIF construction/syntax error, inconsistent or missing data
 8 ALERT type 2 Indicator that the structure model may be wrong or deficient
 7 ALERT type 3 Indicator that the structure quality may be low
 5 ALERT type 4 Improvement, methodology, query or suggestion
 2 ALERT type 5 Informative message, check

It is advisable to attempt to resolve as many as possible of the alerts in all categories. Often the minor alerts point to easily fixed oversights, errors and omissions in your CIF or refinement strategy, so attention to these fine details can be worthwhile. In order to resolve some of the more serious problems it may be necessary to carry out additional measurements or structure refinements. However, the purpose of your study may justify the reported deviations and the more serious of these should normally be commented upon in the discussion or experimental section of a paper or in the "special_details" fields of the CIF. checkCIF was carefully designed to identify outliers and unusual parameters, but every test has its limitations and alerts that are not important in a particular case may appear. Conversely, the absence of alerts does not guarantee there are no aspects of the results needing attention. It is up to the individual to critically assess their own results and, if necessary, seek expert advice.

Publication of your CIF in IUCr journals

A basic structural check has been run on your CIF. These basic checks will be run on all CIFs submitted for publication in IUCr journals (*Acta Crystallographica*, *Journal of Applied Crystallography*, *Journal of Synchrotron Radiation*); however, if you intend to submit to *Acta Crystallographica Section C* or *E* or *IUCrData*, you should make sure that full publication checks are run on the final version of your CIF prior to submission.

Publication of your CIF in other journals

Please refer to the *Notes for Authors* of the relevant journal for any special instructions relating to CIF submission.

PLATON version of 03/05/2019; check.def file version of 29/04/2019

Datablock ZRM180602 - ellipsoid plot

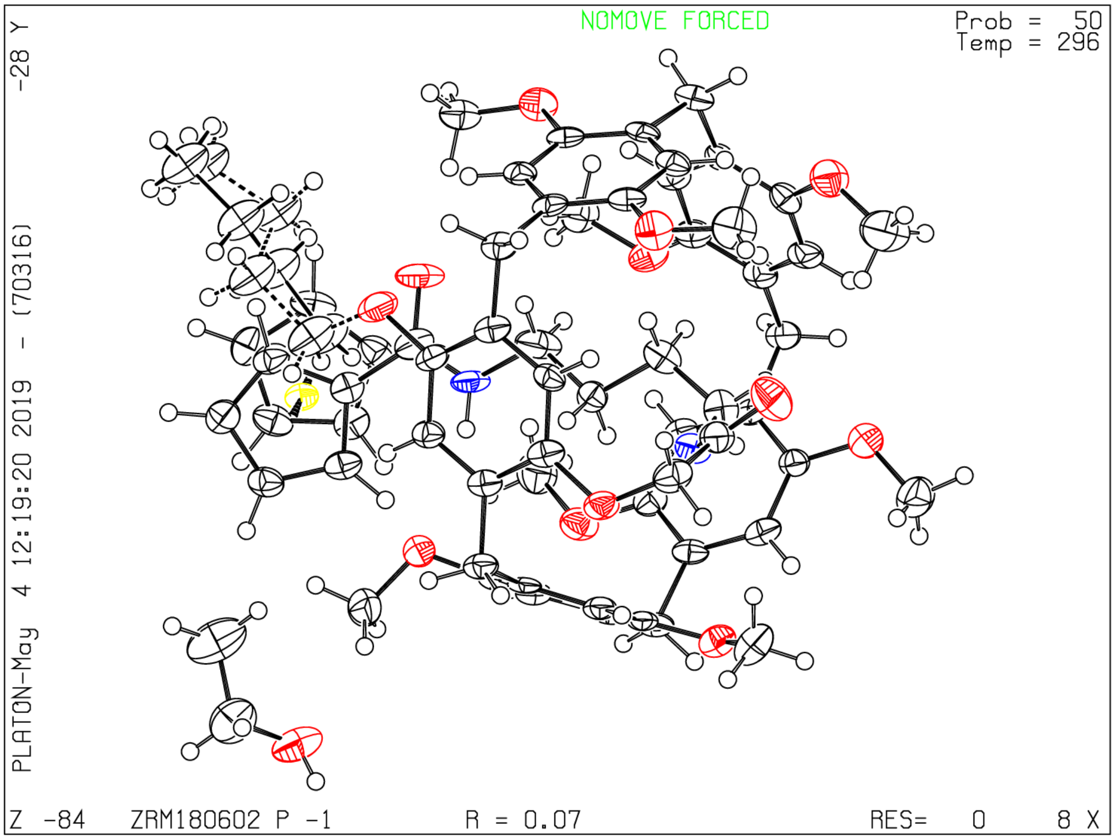

Supplement: Supplementary file 3 [file Data_Sheet_2.PDF]
